# Suppression of *Sproutys* Has a Therapeutic Effect for a Mouse Model of Ischemia by Enhancing Angiogenesis

**DOI:** 10.1371/journal.pone.0005467

**Published:** 2009-05-08

**Authors:** Koji Taniguchi, Ken-ichiro Sasaki, Kousuke Watari, Hideo Yasukawa, Tsutomu Imaizumi, Toranoshin Ayada, Fuyuki Okamoto, Takuma Ishizaki, Reiko Kato, Ri-ichiro Kohno, Hiroshi Kimura, Yasufumi Sato, Mayumi Ono, Yoshikazu Yonemitsu, Akihiko Yoshimura

**Affiliations:** 1 Division of Molecular and Cellular Immunology, Medical Institute of Bioregulation, Kyushu University, Fukuoka, Japan; 2 Department of Surgery and Science, Graduate School of Medical Sciences, Kyushu University, Fukuoka, Japan; 3 Department of Microbiology and Immunology, Keio University School of Medicine, Shinjuku-ku, Tokyo, Japan; 4 Division of Cardiovascular Medicine, Department of Internal Medicine, Kurume University, Kurume, Japan; 5 Department of Pharmaceutical Oncology, Graduate School of Pharmaceutical Sciences, Kyushu University, Fukuoka, Japan; 6 Division of Pathophysiological and Experimental Pathology, Department of Pathology, Graduate School of Medical Sciences, Kyushu University, Fukuoka, Japan; 7 Department of Vascular Biology, Institute of Development, Aging, and Cancer, Tohoku University, Sendai, Japan; 8 Department of Gene Therapy, Chiba University Graduate School of Medicine, Chiba, Japan; 9 Japan Science and Technology Corporation (JST), CREST, Kawaguchi, Japan; University of Giessen Lung Center, Germany

## Abstract

Sprouty proteins (Sproutys) inhibit receptor tyrosine kinase signaling and control various aspects of branching morphogenesis. In this study, we examined the physiological function of Sproutys in angiogenesis, using gene targeting and short-hairpin RNA (shRNA) knockdown strategies. *Sprouty2* and *Sprouty4* double knockout (KO) (DKO) mice were embryonic-lethal around E12.5 due to cardiovascular defects. The number of peripheral blood vessels, but not that of lymphatic vessels, was increased in *Sprouty4* KO mice compared with wild-type (WT) mice. *Sprouty4* KO mice were more resistant to hind limb ischemia and soft tissue ischemia than WT mice were, because *Sprouty4* deficiency causes accelerated neovascularization. Moreover, suppression of *Sprouty2* and *Sprouty4* expression *in vivo* by shRNA targeting accelerated angiogenesis and has a therapeutic effect in a mouse model of hind limb ischemia. These data suggest that Sproutys are physiologically important negative regulators of angiogenesis *in vivo* and novel therapeutic targets for treating peripheral ischemic diseases.

## Introduction

Growth factor-induced signaling by receptor tyrosine kinases (RTKs) plays several essential roles in development and pathogenesis; accordingly, it is tightly controlled by a number of regulatory proteins [1]–[3]. When a ligand binds to an RTK and recruits a Grb2-Sos to the inner surface of a membrane, the Sos protein binds to Ras, causing GDP/GTP exchange and thus activating Ras. Activated Ras recruits Raf to the plasma membrane and activates the Raf/MEK/extracellular signal-regulated kinase (ERK) pathway. Some growth factors, such as vascular endothelial growth factor (VEGF)-A, also activate the Raf/MEK/ERK pathway through the RTK/phospholipase C (PLC)-γ/protein kinase C (PKC) pathway, which is a Ras-independent pathway [4].

Sprouty (Spry) has been genetically identified as an antagonist of fibroblast growth factor (FGF) receptor in tracheal development in *Drosophila*, and is a proven negative regulator of the Ras/Raf/ERK pathway [5], [6]. Four mammalian genes with sequence similarity to *Drosophila Sprouty* (*Sprouty1–4*) have been identified [1], [2]. In addition, we have identified three Sprouty-related proteins known as Spred1–3 (Spreds), in which the C-terminal cysteine-rich domain found in Sprouty proteins (Sproutys) is conserved [7], [8]. Since loss-of-function mutations of the *SPRED1* gene have been found in human neuro-cardio-facial-cutaneous (NCFC) syndromes [9], and since these syndromes are caused by dysregulation of the Ras-ERK pathway, we conclude that SPRED1 is a negative regulator of RTK-mediated Ras/ERK activation.

In the development of the cardiovascular system of *Drosophila*, as in the tracheal system, the formation of new blood vessels from preexisting ones (angiogenesis) involves the sprouting of endothelial cells out of an epithelial layer and the branching of tubular structures [10]. In the adult, angiogenesis only takes place during the female reproductive cycle, during wound healing, and in pathological situations, including tumor growth, diabetic retinopathy, arthritis, atherosclerosis, and psoriasis [10], [11]. Angiogenesis is tightly regulated by a balance between inducing and inhibitory signals [12]. Growth factors, such as VEGF-A, basic FGF (bFGF), and angiopoietins, positively regulate angiogenesis by binding to their cognate RTKs and thus inducing endothelial cell proliferation, migration, differentiation, and survival [12], [13]. In addition, sphingosine-1-phosphate (S1P), which activates GPCRs, has also been implicated in angiogenesis [14]. In contrast, proteins that negatively regulate angiogenesis by specifically blocking RTK signaling are less well characterized.

Previous studies have demonstrated that overexpression of Sproutys inhibits VEGF-A- and bFGF-induced endothelial cell proliferation and differentiation *in vitro* as well as branching and sprouting of small vessels *in vivo* [15], [16]. Moreover, Sprouty4 suppresses VEGF-A/VEGF receptor (VEGFR)-2 signaling *in vitro* [17]–[19]. We also know that Spreds, in contrast, inhibit VEGF-C signaling, which is important in lymphangiogenesis, and that *Spred1/Spred2* double knockout (KO) (DKO) mice show abnormal lymphatic development [18]. Yet the physiological role of Sproutys in angiogenesis and lymphangiogenesis remains to be elucidated.

In this study we investigated the physiological function of Sproutys in angiogenesis by performing knockout and knockdown analyses of *Sproutys*. We showed that Sproutys are negative regulators for angiogenesis rather than lymphangiogenesis *in vivo*. Moreover, *Sprouty4* KO mice were more resistant to hind limb ischemia and soft tissue ischemia than wild-type (WT) mice were, and *in vivo* shRNA targeting *Sprouty2* and *Sprouty4* accelerated angiogenesis in a mouse model of hind limb ischemia. These data suggest that Sprouty2 and Sprouty4 are important negative regulators of angiogenesis *in vivo* that could be new therapeutic targets for ischemic diseases.

## Results

### Increased developmental angiogenesis in *Sprouty*-deficient mice

**Figure 1:**
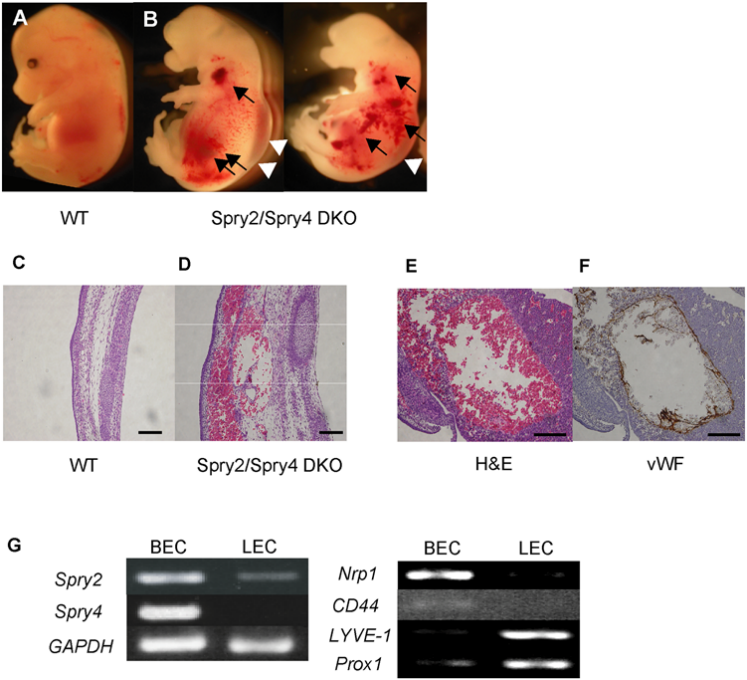
Characterization of *Sprouty2/Sprouty4* DKO mice. (A, B) Gross appearance of wild-type (WT) (A) and *Sprouty2/Sprouty4* DKO (B) embryos at embryonic day 12.5. The arrow and arrowheads indicate hemorrhage and edema, respectively. (C, D) Hematoxylin-eosin (H&E) staining of sections of WT (C) and *Sprouty2/Sprouty4* DKO (D) skin. (E, F) H&E staining and immunohistochemical staining with von Willebrand factor (vWF) of sections of hepatic hemangiomas in *Sprouty2/Sprouty4* DKO liver. vWF was used as a blood vessel marker. (G) Expression of *Sproutys* in endothelial cells. About 5.0×10^4^ BECs and LECs were FACS-sorted at embryonic day 14.5, and were used for RT-PCR analysis. *GAPDH* served as a loading control. Good separation of BECs and LECs was confirmed by BEC markers (*Nrp1*, *CD44*) and LEC markers (*LYVE1*, *Prox1*). Scale bars (C–F): 100 µm.

Overexpression studies suggest that Sprouty2 and Sprouty4 possess similar negative effects on RTK-mediated ERK activation [20]. To define the overlapping functions of Sprouty2 and Sprouty4, we generated *Sprouty2/Sprouty4* DKO mice. *Sprouty2/Sprouty4* DKO mice were embryonic-lethal by embryonic day 12.5 and showed very severe defects in craniofacial and limb morphogenesis [21]. They also showed very severe subcutaneous hemorrhage, edema (Fig. 1A–D), and multiple hepatic hemangiomas (Fig. 1E,F), which suggested that they had cardiovascular defects as well. We next investigated the expression pattern of *Sprouty2* and *Sprouty4* in endothelial cells during embryonic development, and found that *Sprouty2* and *Sprouty4* were more highly expressed in blood endothelial cells (BECs) than in lymphatic endothelial cells (LECs) (Fig. 1G).

**Figure 2:**
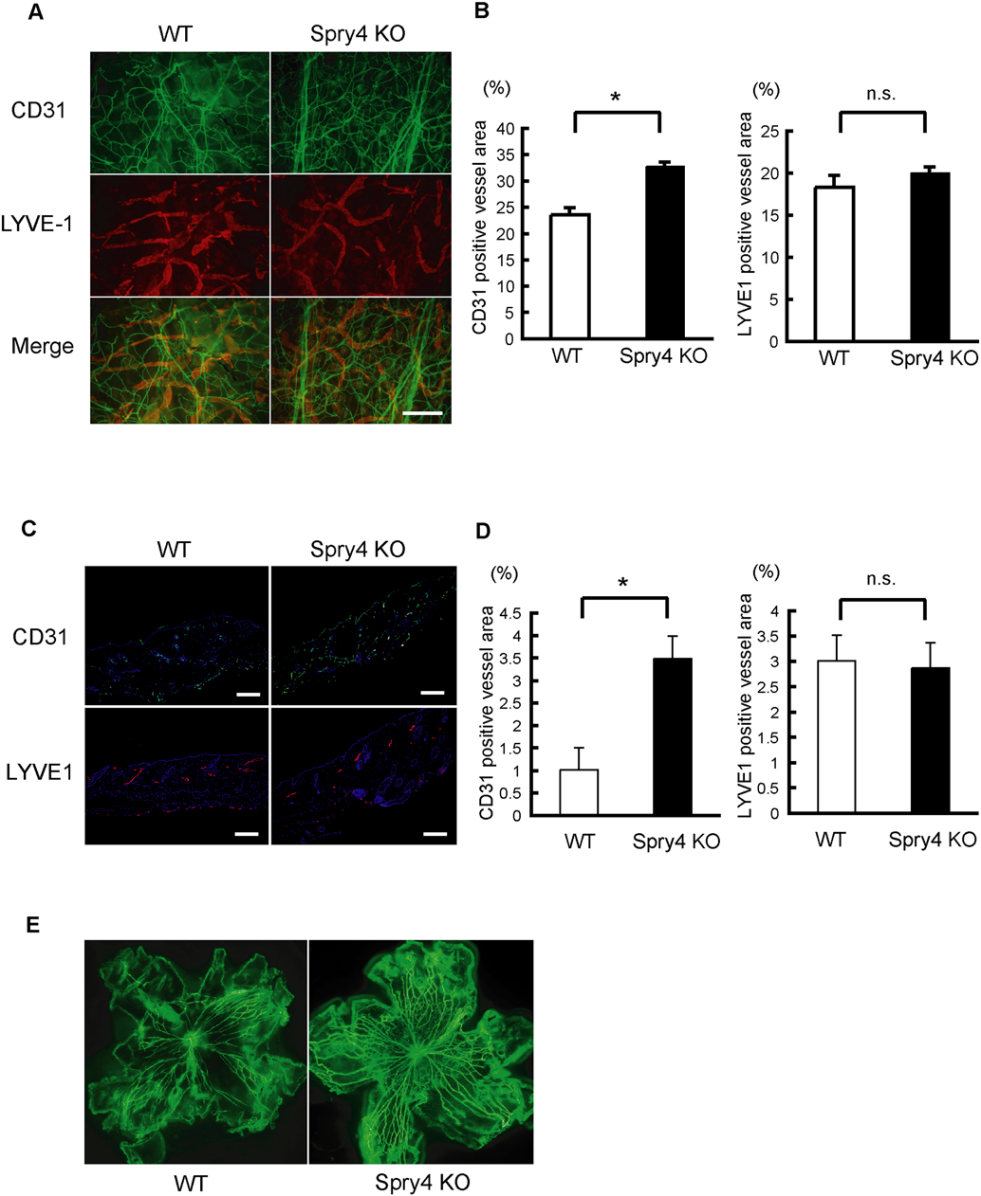
Blood and lymphatic vessels of *Sprouty4* single KO mice. (A) Blood vessels (green) and lymphatic vessels (red) in the ears of WT and *Sprouty4* KO mice (8 weeks old) were analyzed by whole-mount immunohistochemical staining with anti-PECAM-1/CD31Ab and anti-LYVE-1 Ab, respectively. (B) CD31-positive vessel area or LYVE1-positive area was quantified. Data shown are means±SEM. *: *P*<0.05. (C) Blood vessels (green) and lymphatic vessels (red) in the dorsal skin of WT and *Sprouty4* KO mice (8 weeks old) were analyzed by immunohistochemical staining with anti-PECAM-1/CD31Ab and anti-LYVE-1 Ab, respectively. Nuclei were stained with Hoechst 33342 dye (Blue). (D) CD31-positive vessel area or LYVE1-positive area was quantified. Data shown are means±SEM. *: *P*<0.05. (E) FITC-dextran-perfused flat-mounted retinal samples of WT and *Sprouty4* KO mice at postnatal day 3. Scale bars (A, C): 100 µm.

This discovery led us to examine vascularization in adult *Sprouty4* single KO mice in detail, although *Sprouty4* single KO mice showed no obvious vascular phenotype [21]. *Sprouty4* single KO mice exhibited more vascular networks of blood vessels in the ear than WT mice did (Fig. 2A,B). Similarly, more vascular networks of blood vessels in the ear were observed in *Sprouty2* single KO mice than in WT mice (data not shown). The numbers of blood vessels in the skin were also increased in *Sprouty4* KO mice (Fig. 2C,D). Lymphatic vessel networks, on the other hand, were present at the same frequency in these *Sprouty4* KO mice as in WT mice (Fig. 2A–D). Retinal vasculature is a good model system for the study of general blood vessel development [22]. Vascular development in the early embryo is difficult to observe, but the murine retinal vascular system develops after birth and is therefore easier to examine. We compared flat-mounted retinas from WT and *Sprouty4* KO mice at postnatal day (PD) 3 after injecting FITC-dextran (Fig. 2E). As the image clearly shows, retinal angiogenesis was enhanced in *Sprouty4* KO mice compared to WT mice.

These data suggest that, in contrast to Spred1 and Spred2, which are important negative regulators of developmental lymphangiogenesis rather than developmental angiogenesis, as previously reported [18], Sprouty2 and Sprouty4 are important negative regulators of developmental angiogenesis rather than developmental lymphangiogenesis.

### *Sprouty4* KO mice are more resistant to ischemia

Next, we sought to investigate the effect of *Sprouty4* deficiency in the ischemia-induced angiogenesis model, an adult neovascularization assay which is useful for quantifying neovascularization in *Sprouty4* KO mice. We used mouse models of hind limb ischemia [23] and soft tissue ischemia [24]. We used *Sprouty4* KO mice, since *Sprouty4* KO mice can survive much longer than *Sprouty2* KO mice [21], [25].

**Figure 3:**
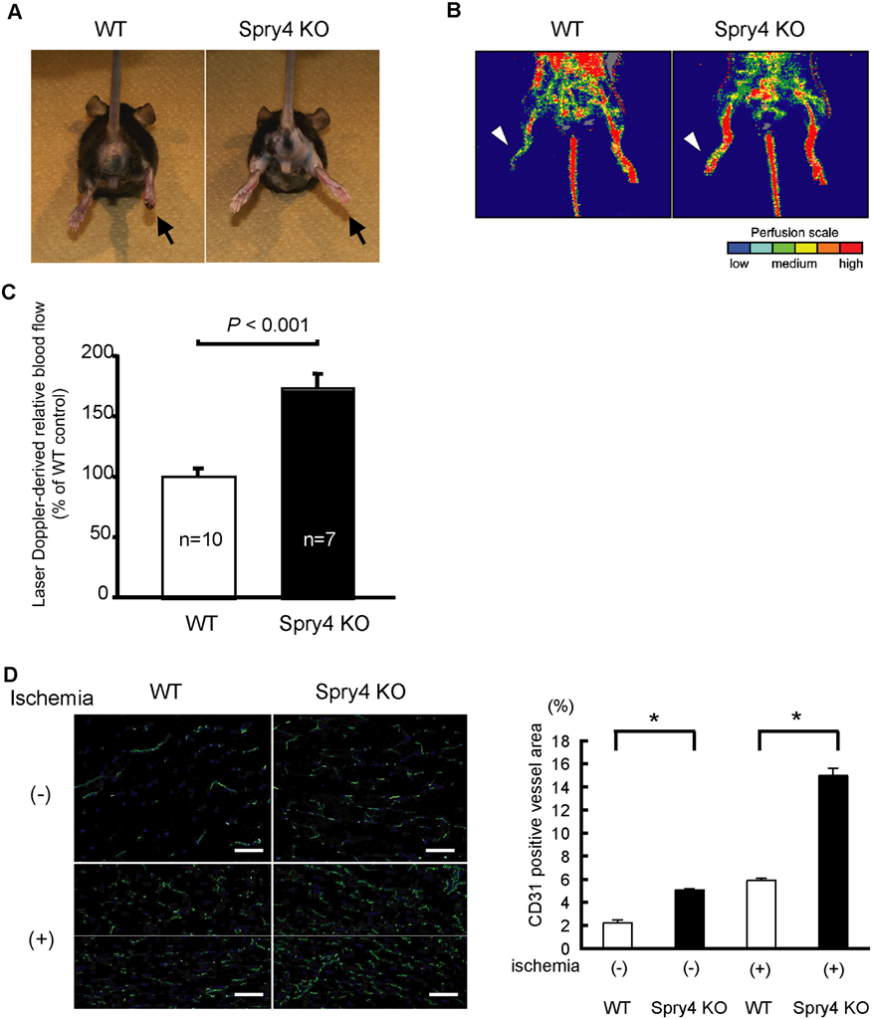
*Sprouty4* KO mice are more resistant in a hind-limb ischemia model. (A) Representative photos of ischemic limbs, indicated by arrows. (B) Representative laser Doppler images for each group are depicted. Arrowheads indicate ischemic limbs. The interval of low perfusion is displayed as dark blue; the highest perfusion interval is displayed as red. (C) Recovery of limb perfusion in WT (n = 10) and *Sprouty4* KO (n = 7) mice after hind limb ischemia as assessed by laser Doppler blood flow analysis on day 14. Data shown are means±SD. *: *P*<0.001. (D) Blood vessels (green) in the non-ischemic or ischemic adductor muscles of male WT and *Sprouty4* KO mice (8–10 weeks old) were analyzed by immunohistochemical staining with anti-PECAM-1/CD31Ab. Nuclei were stained with Hoechst 33342 dye (blue). The CD31-positive vessel area was quantified. Data shown are means±SEM. *: *P*<0.05. Scale bars: (D) 100 µm.

The former model revealed that *Sprouty4* KO mice were more resistant to hind limb ischemia than WT mice were (Fig. 3A). *Sprouty4* KO mice showed a significantly elevated recovery of limb perfusion after induction of hind limb ischemia as compared with WT mice, and the ischemic/non-ischemic leg perfusion ratio was much more favorable in *Sprouty4* KO mice than in WT mice (*P*<0.001) (Fig. 3B,C). Additionally, angiogenesis in the ischemic hind limb was significantly increased in *Sprouty4* KO mice compared with WT mice (Fig. 3D).

**Figure 4:**
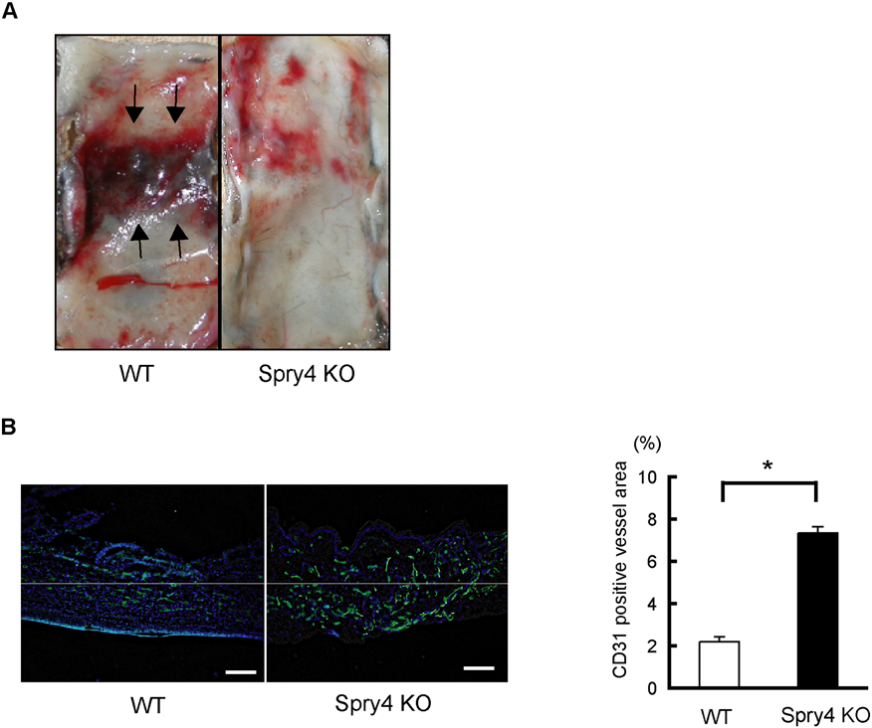
*Sprouty4* KO mice are also more resistant in a soft tissue ischemia model. (A) Representative photos of ischemic dorsal skin of male WT and *Sprouty4* KO mice (8–10 weeks old). Arrows indicate necrotic skin. (B) Left: Blood vessels (green) in the ischemic dorsal skin of male WT and *Sprouty4* KO mice were analyzed by immunohistochemical staining with anti-PECAM-1/CD31Ab. Nuclei were stained with Hoechst 33342 dye (blue). Right: The CD31-positive vessel area was quantified. Data shown are means±SEM. *: *P*<0.05. Scale bars (B): 100 µm.

The latter model was induced by creating lateral skin incisions on the dorsal surfaces of mice. The overlying skin was undermined, and a silicone sheet was inserted into each mouse to separate the skin from the underlying tissue bed. As a result, the most central portion of skin underwent the most severe ischemic insult, which, in WT mice, ultimately led to the absence of flow and necrosis in the central portion of the skin (Fig. 4A). In *Sprouty4* KO mice, however, angiogenesis in the dorsal skin was significantly increased compared to that in WT mice (Fig. 4B). As a result, *Sprouty4* KO mice were more resistant to soft tissue ischemia than WT mice were, and gross evidence of necrosis in the dorsal skin was more evident in WT mice than in *Sprouty4* KO mice (100% and 16.7% in WT mice and *Sprouty4* KO mice, respectively, n = 6) (Fig. 4A).

These data show that *Sprouty4* KO mice exhibit enhanced neovascularization in ischemia-induced models.

### Increased ischemia-induced neovascularization by *in vivo* shRNA targeting *Sproutys*

The increased vessel density in the skin and muscles of untreated *Sprouty4* KO mice (Fig. 2C,D, Fig. 3D) provides them with elevated blood-vessel area in these regions, which is partially responsible for their increased resistance to ischemia.

**Figure 5:**
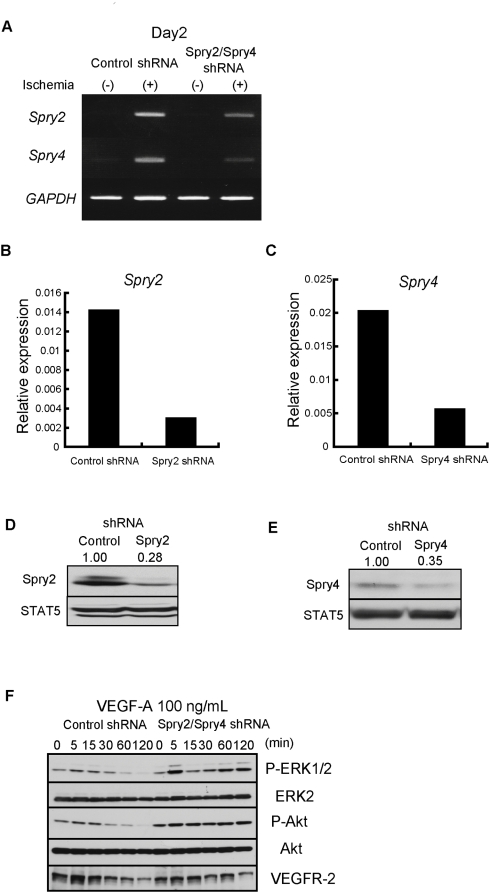
*In vivo* effects of shRNA targeting *Sprouty2* and *Sprouty4*. (A) The *in vivo* effects of shRNA plasmids targeting *Sproutys* in the hind limb model were evaluated by RT-PCR analysis. (B, C) Real-time PCR analysis of *Sprouty2* (B) or *Sprouty4* (C) mRNA expression in MEFs stably infected with control retroviruses and retroviruses expressing either *Sprouty2* shRNA (B) or *Sprouty4* shRNA (C). (D, E) Western blot analysis of protein extracts from MEFs stably infected with control retroviruses and retroviruses expressing either *Sprouty2* shRNA (D) or *Sprouty4* shRNA (E). The relative intensities of Sprouty2 and Sprouty4 bands normalized by STAT5 expression levels are shown above. (F) Effect of both *Sprouty2* and *Sprouty4* knockdown on ERK and Akt activities. MEFs stably expressing VEGFR-2 were infected with control retroviruses and retroviruses expressing *Sprouty2/Sprouty4* shRNA, and stimulated with 100 ng/mL VEGF-A. Cell extracts were immunoblotted with the indicated antibodies.

To investigate *in vivo* the efficiency of down-regulating *Sproutys* as therapy for peripheral ischemic diseases, we administered ischemia treatment to the hind limbs of C57BL/6J mice, then injected shRNA targeting *Sprouty2* and *Sprouty4*. We suppressed both *Sprouty2* and *Sprouty4* simultaneously, because both of the mRNAs increased during hind limb ischemia (Fig. 5A), because the phenotype of *Sprouty2/Sprouty4* DKO mice demonstrated a redundant role of *Sprouty2* and *Sprouty4* in angiogenesis (Fig. 1), and because we have not found any functional differences between Sprouty2 and Sprouty4 *in vitro* [20]. The shRNA plasmid targeting *Sprouty2* and *Sprouty4* efficiently suppressed the expression levels of endogenous Sprouty2 and Sprouty4, respectively, in both real-time PCR (Fig. 5B,C) and Western blot (Fig. 5D,E) analysis. The shRNA plasmids targeting both *Sprouty2* and *Sprouty4* enhanced VEGF-A-induced ERK and Akt activation *in vitro* in mouse embryonic fibroblasts (MEFs), which stably expressed VEGFR-2 (Fig. 5F). We used MEFs because it has been very difficult to introduce shRNA into primary murine endothelial cells *in vitro*. Although we confirmed that shRNA against Sprouty2 or Sprouty4 alone enhanced VEGF-A-induced ERK and Akt activation (data not shown), we observed much stronger effect by the combination of Sprouty2 and Sprouty4 shRNAs. Thus we decide to use combination of both Sprouty2 and Sprouty4 shRNAs for further experiments.

**Figure 6:**
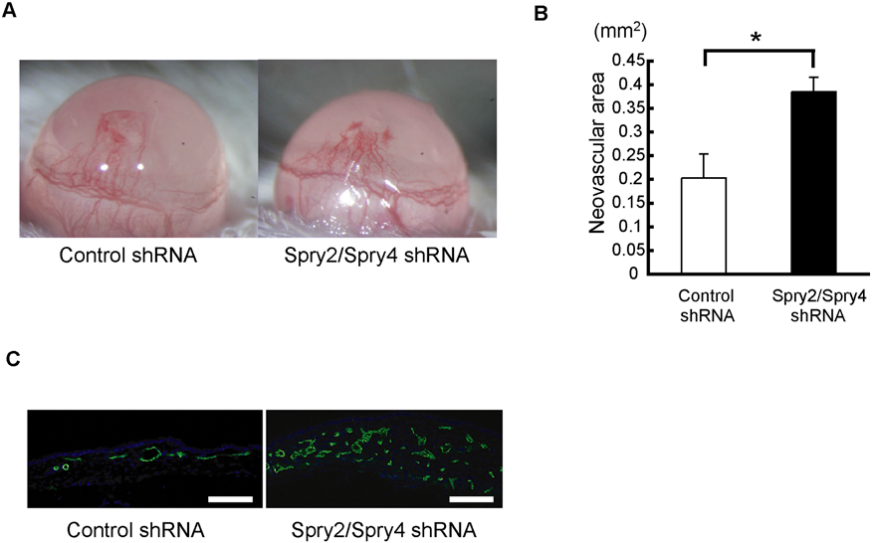
*In vivo* effects of shRNA targeting *Sprouty2* and *Sprouty4* in corneal micropocket assay. (A) Corneal neovascularization was induced by mouse VEGF-A (200 ng) on day 12 after hydron pellets had been implanted into male BALB/c mouse corneas. After implantation, 10 µg shRNA plasmids per eye were delivered by subconjunctival injection. Representative photos are shown. (B) Quantitative analysis of neovascularization on day 12. Areas are expressed in mm^2^. Bars show the mean±SEM (n = 5). *: *P*<0.05. (C) Sections of corneas implanted with VEGF-A stained by anti-PECAM-1/CD31Ab on day 12. Scale bars (C): 100 µm.

First, we showed that injection of shRNA plasmids targeting *Sprouty2* and *Sprouty4* significantly enhanced corneal neovascularization induced by VEGF-A, compared to control shRNA plasmids in a corneal micropocket assay (Fig. 6A–C). These data indicate that *Sprouty2* and *Sprouty4* shRNA plasmids can efficiently suppress the expression levels of Sprouty2 and Sprouty4 and block the effect of endogenous Sprouty2 and Sprouty4 *in vitro* and *in vivo*.

**Figure 7:**
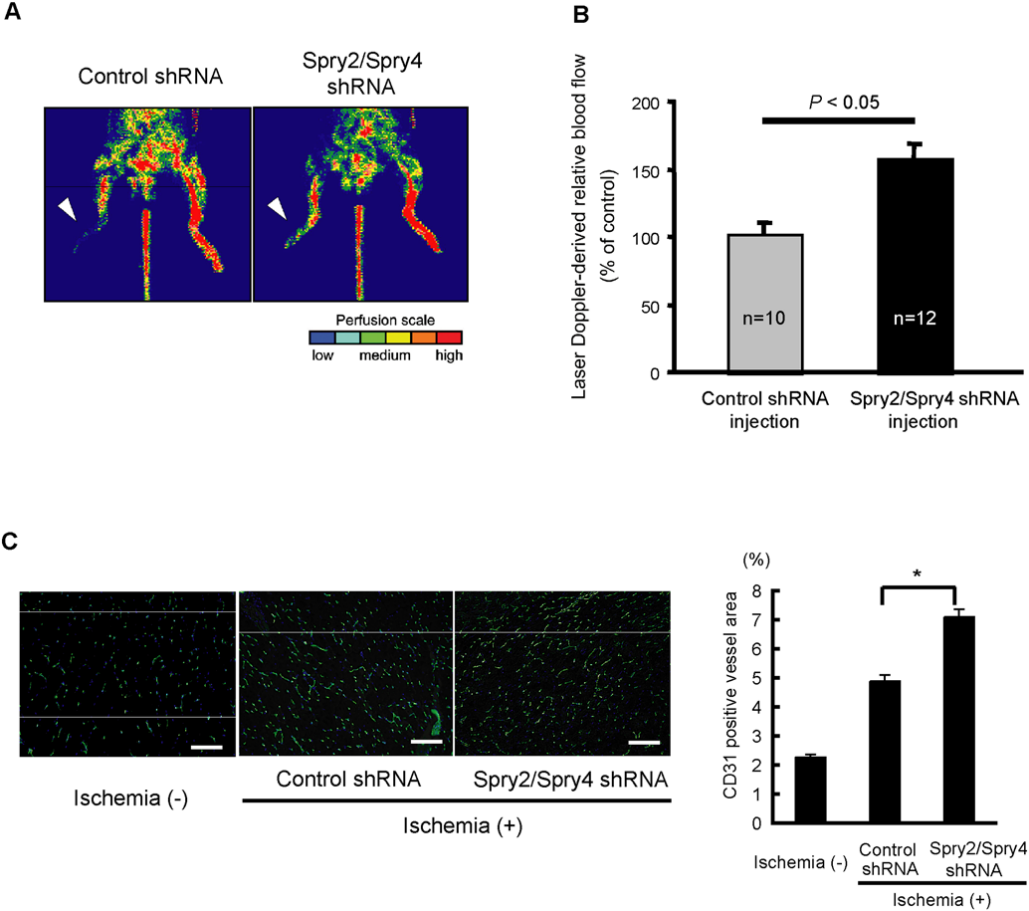
Increased ischemia-induced angiogenesis by *in vivo* shRNA targeting *Sprouty2 and Sprouty4*. (A) Representative laser Doppler images for each group are depicted. Arrowheads indicate ischemic limbs. The interval of low perfusion is displayed as dark blue; the highest perfusion interval is displayed as red. (B) Recovery of limb perfusion in C57BL/6J mice (8 weeks old) injected with the control shRNA (n = 10) or *Sprouty2/Sprouty4* shRNA vectors (n = 12) after hind limb ischemia as assessed by laser Doppler blood flow analysis on day 14. Data shown are means±SD. *: *P*<0.05. (C) Blood vessels (green) in the non-ischemic or ischemic adductor muscle injected with the control shRNA or *Sprouty2/Sprouty4* shRNA vectors stained with anti-PECAM-1/CD31Ab. Nuclei were stained with Hoechst 33342 dye (blue). The CD31-positive vessel area was quantified. Data shown are means±SEM. *: *P*<0.05. Scale bars (C): 100 µm.

Next, we investigated whether *Sprouty2* and *Sprouty4* shRNA plasmids were effective in a mouse model of hind limb ischemia. Upon injection to the ischemic adductor muscle, *Sprouty2* and *Sprouty4* shRNA plasmids reduced *Sprouty2* and *Sprouty4* expression *in vivo* (Fig. 5A). *Sprouty2* and *Sprouty4* shRNA plasmids induced a significantly elevated recovery of limb perfusion after induction of hind limb ischemia as compared with control shRNA plasmids, and markedly improved the ischemic/non-ischemic leg perfusion ratio (*P*<0.05) (Fig. 7A,B). shRNA plasmids targeting *Sprouty2* and *Sprouty4* also increased capillary density compared with control shRNA plasmids (Fig. 7C). Our data clearly demonstrate that Sprouty2 and Sprouty4 negatively regulate angiogenesis *in vivo* and would make good therapeutic targets for peripheral ischemic diseases.

## Discussion

In this study, we investigated the physiological function of Sproutys in angiogenesis by performing a knockout and knockdown analysis of *Sproutys*. In contrast to Spred1 and Spred2, which regulate developmental lymphangiogenesis, Sprouty2 and Sprouty4 are important negative regulators of developmental angiogenesis *in vivo*. We found that the amounts of blood vessels in *Sprouty4* KO mice are increased in all tissues we investigated. So we think that all peripheral blood vessels are increased in *Sprouty4* KO mice. *Sprouty4* deficiency enhanced ischemia-induced angiogenesis in mouse models of hind limb ischemia and soft tissue ischemia. Moreover, the suppression of *Sprouty2* and *Sprouty4* expression *in vivo* by shRNA targeting had a therapeutic effect in our model of hind limb ischemia, indicating that Sproutys should be novel therapeutic targets for treating peripheral ischemic diseases.

The roles of Sprouty and Spred proteins during gastrulation in *Xenopus tropicalis* have been compared elsewhere [26]. Spred proteins preferentially inhibit the Ras/ERK cascade that directs mesoderm formation, whereas Sprouty proteins block the Ca^2+^ and PKCδ signals required for morphogenetic movements during gastrulation. Thus, the expression of *Sprouty* and *Spred* genes at specific times during gastrulation might redirect FGF signals toward mesoderm formation or morphogenesis, respectively [26]. In mammalian development, *Sproutys* are expressed mainly in blood endothelial cells, while *Spreds* are expressed mainly in lymphatic endothelial cells (Fig. 1G and Ref. [18]). In overexpression experiments, while Sproutys can inhibit VEGF-A signaling but not VEGF-C signaling, Spreds can suppress both types (Taniguchi K., unpublished data and Ref. [18]). Indeed, microRNA-126 deletion suppresses VEGF-A-induced ERK activation in endothelial cells and angiogenesis through the increase of Spred1 [27]–[29]. However, the effect of *Sprouty* deletion is more specific to VEGF-A signaling than to VEGF-C signaling, while the effect of *Spred* deletion is more specific to VEGF-C signaling than to VEGF-A signaling (Taniguchi K., unpublished data and Ref. [18]). In fact, *Sprouty2* and *Sprouty4* single-deficient mice showed defects of blood vessels rather than lymphatic vessels, while *Spred1/Spred2* DKO mice showed abnormal lymphatic vessel development and nearly normal blood vessel development (Fig. 2 and Ref. [18]). In addition to this difference in expression, these results suggest that Sproutys and Spreds might have different functions in endothelial cells. VEGF-A/VEGFR-2 signaling is Ras-independent and PLC-γ/PKC-dependent, while VEGF-C/VEGFR-3 signaling is Ras-dependent and PKC-independent (Taniguchi K., unpublished data and Ref. [4]). Therefore, drawing an analogy from the different functions of Sproutys and Spreds in *Xenopus tropicalis*, we propose that Sproutys inhibit PLC-γ/PKC-dependent VEGF-A signaling and angiogenesis, while Spreds inhibit Ras-dependent VEGF-C signaling and lymphangiogenesis.

Although, in ischemia-induced angiogenesis, VEGF-A is thought to be the primary angiogenesis-stimulating factor [30], angiogenesis is the more complex process, as it is triggered not only by VEGF-A but also by bFGF, S1P, angiopoietins, and others [12]–[14]. In fact, it is reported that *bFGF* gene therapy is effective to treat critically ischemic limb [31]. It is already known that Sproutys can inhibit various RTK signals [1], [2]. We have also shown that loss of *Sprouty* expression results in hyperactivation of VEGF-A and bFGF signaling as well as S1P and LPA signaling (Fig. 5F, Taniguchi K., unpublished data and Ref. [19] and [21]). It is reported that *in vivo* shRNA targeting *SHP-1* also accelerated angiogenesis in a rat model of hind limb ischemia [32]. While SHP-1 inhibits only RTK signals, Sproutys suppress both RTK and GPCR signals. Thus the suppression of *Sproutys* could be beneficial.

Inhibition of negative feed-back loops leading to profound and long term activation of signals often lead to a dysregulation of neovascularisation since the overshooting response is inducing immature vessels. However, excessive sprouting in response to inhibition of Sproutys results in the formation of mature vessels. Angiogenesis is a complex process that includes the recruitment and proliferation of various cells, such as endothelial cells, mural cells [smooth muscle cells (SMC) and pericytes], endothelial progenitor cells (EPCs) and others. It is reported that *Sprouty*-family genes are expressed in both endothelial cells and smooth muscle cells [33], and we have confirmed that *Sprouty/Spred* family genes are also expressed in bone marrow (Taniguchi K., unpublished data). It is possible that Sproutys function not only in endothelial cells, but also in mural cells, EPCs or myeloid cells, and that enhanced angiogenesis of mature vessels in *Sprouty4* KO mice and the results of our experiments with *in vivo* shRNA targeting *Sproutys* are partially dependent on the enhanced function or the increased number of mural cells, EPCs or myeloid cells. Moreover, it is possible that Sproutys are also associated with angiopoietins signals, which are important for the maturation of blood vessels. Further study is necessary to investigate these possibilities.

*Sprouty4* KO mice were more resistant to ischemia than WT mice were in mouse models of ischemia (Fig. 3, Fig. 4), and neovascularization induced by a tumor transplantation model was also accelerated by *Sprouty4* deficiency (Taniguchi K., unpublished data). Moreover, *in vivo* shRNA targeting *Sprouty2* and *Sprouty4* accelerated angiogenesis in a mouse model of hind limb ischemia (Fig. 7). In this study, muscle tissue injected with the *Sproutys* shRNA vectors exhibited a significant decrease in *Sproutys* transcripts (Fig. 5A). This knockdown efficiency may be due to the fact, in skeletal muscle, the efficiency of intramuscular gene transfer has been shown to be augmented from five- to seven-fold when the injected muscle is ischemic [34]. The present study is the first to uncover these significant implications for gene therapy using the *Sprouty2* and *Sprouty4* shRNA vectors for the treatment of peripheral ischemic diseases. The fact that Sproutys exhibit such broad suppression activity, inhibiting a wide variety of angiogenic factors and cells, indicates that the suppression of *Sproutys* must enhance neovascularization.

In conclusion, Sproutys are physiologically important regulators of angiogenesis *in vivo* and may be useful as new therapeutic targets for peripheral ischemic diseases.

## Methods

### Mice

*Sprouty2* KO mice and *Sprouty4* KO mice have been described previously [21], [25]. *Sprouty2* KO mice and *Sprouty4* KO mice were generated as 129/C57BL/6J mixed background, and then backcrossed into C57BL/6J at least five times. Gender-matched, WT littermates were used as controls. All experiments using these mice were approved by and performed according to the guidelines of the Animal Ethics Committee of Kyushu University, Fukuoka, Japan.

### Cell culture

Primary mouse embryonic fibroblasts (MEFs) were prepared, as previously described [21]. MEFs were cultured in Dulbecco's modified Eagle's medium (DMEM) (Gibco, Grand Island, NY, USA) supplemented with 10% fetal bovine serum, penicillin and streptomycin. To generate MEFs stably expressing VEGFR-2 or shRNAs, MEFs were infected with the retroviruses produced by Plat-E, packaging cell line, transfected with pMX-VEGFR-2 or shRNA plasmids, and then the infected cells were selected with 1 µg/ml puromycin (Invivogen, San Diego, CA, USA), as previously described [18], [35].

### Antibodies and reagents

Antibodies used in this experiment were as follows: anti-phospho-ERK1/2 (#9106), anti-phospho-Akt (#4058), and anti-Akt (#9272) (Cell Signaling Technology, Danvers, MA, USA); anti-Sprouty4 (H-100), anti-VEGFR-2 (A-3) and anti-ERK2 (C-14) (Santa Cruz Biotechnology, Santa Cruz, CA, USA); anti-Sprouty2 (ab50317) (Abcam, Cambridge, MA, USA); and anti-vWF (DAKO, Glostrup, Denmark); Mouse VEGF-A was purchased from R&D Systems (Minneapolis, MN, USA). Human VEGF-A was purchased from PeproTech (London, UK).

### RT-PCR and real-time PCR analysis

The cells or tissues were lysed in RNAiso (TAKARA BIO, Shiga, Japan) for RNA preparation. Total RNA was isolated through fluorescence activated cell sorting (FACS), which sorted about 5.0×10^4^ BECs and LECs at embryonic day 14.5, as previously reported [18]. Good separation of BECs and LECs was confirmed by BEC markers (*Nrp1*, *CD44*) and LEC markers (*LYVE-1*, *Prox1*). Total RNA was reverse transcribed using the High Capacity cDNA Reverse Transcription Kit (Applied Biosystems, Foster City, CA, USA), and the product was used for further analysis. PCR products were separated on 2.0% agarose gel stained with ethidium bromide. The expression level of *GAPDH* was evaluated as an internal control. The primer sequences for RT-PCR were as follows: *Sprouty2*-F, 5′- TTTTAATCCACCGATTGCTTGG-3′; *Sprouty2*-R, 5′-GCTGCACTCGGATTATTCCATC-3′; *Sprouty4*-F, 5′-CAGCTCCTCAAAGACCCCTAGAAGC-3′; *Sprouty4*-R, 5′-GTGCTGCTACTGCTGCTTACAGAGC-3′; *GAPDH*-F, 5′-ACCACAGTCCATGCCATCAC-3′ and *GAPDH*-R 5′-TCCACCACCCTGTTGCTGTA-3′. The primer sequences for BEC and LEC markers were described elsewhere [36]. Real-time PCR was performed on cDNA samples using an ABI 7000 Sequence Detection System (Applied Biosystems) with the SYBR Green system (Applied Biosystems). The relative quantitation value is expressed as 2^−*Ct*^, where *Ct* is the difference between the mean *Ct* value of triplicates of the sample and of the endogenous *GAPDH* control. The primer sequences for real-time PCR were as follows: *Sprouty2*-F, 5′-ATAATCCGAGTGCAGCCTAAATC-3′; *Sprouty2*-R, 5′-CGCAGTCCTCACACCTGTAG-3′; *Sprouty4*-F, 5′-CGACCAGAGGCTCCTAGATCA-3′; *Sprouty4*-R, 5′-CAGCGGCTTACAGTGAACCA-3′; *GAPDH*-F, 5′-TGTGTCCGTCGTGGATCTGA-3′ and *GAPDH*-R 5′-CCTGCTTCACCACCTTCTTGA-3′.

### Western blot analysis

Western blot analysis was performed as described previously [18]. MEFs were lysed in lysis buffer (50 mM Tris-HCl, pH 7.6, 150 mM NaCl, 1% Nonidet P-40, 1 mM sodium vanadate) supplemented with protease inhibitors (Nacalai tesque, Kyoto, Japan). About 20 µg of proteins were separated by SDS-PAGE and transferred to Immobilon-P nylon membranes (Millipore, Bedford, MA, USA).

### Immunohistochemistry

Whole-mount immunohistochemistry of adult ears or immunohistochemistry of adult skin was performed with 1∶200 diluted anti-PECAM-1/CD31 (MEC13.3, BD Pharmingen, Franklin Lakes, NJ, USA) or anti-LYVE-1 antibody (Acris Antibodies, Hiddenhausen, Germany) essentially as described previously [18].

### Retinal angiography

Flat-mounted retinas were evaluated using fluorescein–dextran angiography as described elsewhere [22]. The mice were deeply anesthetized and a 0.03 ml/g body weight 50 mg/mL solution of 2×10^6^ molecular weight FITC–dextran (Sigma, St Louis, MO, USA) was perfused through the left ventricle. The eyes were enucleated and fixed in 4% paraformaldehyde for at least 3 h. The corneas and lenses were then removed, and the peripheral retinas were dissected and flat-mounted on microscope slides for examination under a fluorescence microscope.

### Vessel quantitative analysis

The vascular area in the ear, skin or muscle was quantified as a PECAM-1/CD31-positive area from ten ×10 micrographs, using Image J software (http://rsb.info.nih.gov), as described elsewhere [37]. LYVE-1-positive vessels in the skin were quantified in a similar manner.

### RNAi-mediated knockdown

The mammalian expression vector pSUPER.retro.puro (Oligoengine, Seattle, WA, USA) was used for expression of shRNA targeting murine *Sprouty2*. The sequence of the *Sprouty2* shRNA is 5′-GCCGGGTTGTCGTTGTAAA-3′ and corresponds to nucleotides 1150–1168 of *mSprouty2*. Murine *Sprouty4* shRNA (29-mer) and control plasmids were purchased from Origene (Rockville, MD, USA) and used according to the manufacturer's protocols. The specificity of *Sprouty2* and *Sprouty4* knockdown was confirmed by real-time PCR or immunoblotting of whole-cell lysates of MEFs with anti-Sprouty2 and anti-Sprouty4 antibodies, respectively. The relative intensities of Sprouty2 or Sprouty4 band were normalized by STAT5 expression using Image J software, as previously described [9], [35].

### In vivo models of ischemia

A hind limb ischemia model was performed as previously described [23], [38]. Male WT and *Sprouty4* KO mice (8–10 weeks old) and male C57BL/6J mice (8 weeks old) were used for a hind limb ischemia model. The proximal portion of the right femoral artery including the superficial and the deep branch and the distal portion of the saphenous artery were occluded with an electrical coagulator. After 2 weeks, we determined the ischemic (right)/nonischemic (left) limb blood flow ratio by using a laser Doppler blood flow imager (Laser Doppler Perfusion Imager System, moorLDI-Mark 2; Moor Instruments, Wilmington, DE, USA). Before initiating scanning, mice were placed on a heating pad at 37°C to minimize variations in their body temperatures. Calculated perfusion is expressed as the ratio of ischemic to nonischemic hind limb perfusion. At 2 wk after femoral resection, adductor muscles from the ischemic and control limbs were embedded in OCT compound. Eight-micron sections were stained with anti-PECAM-1/CD31 antibody (BD Pharmingen) and anti-rat Ig secondary antibody. For *in vivo* shRNA targeting *Sprouty2* and *Sprouty4*, a hind limb ischemia model was performed. Immediately after ischemia was induced, either a total of 40 µg *Sproutys* shRNA vectors (20 µg *Sprouty2* shRNA vector and 20 µg *Sprouty4* shRNA vector) or a quantity of control shRNA vectors was injected into five different sites in the adductor muscle of each anesthetized mouse [32]. A model of soft tissue ischemia similar to one described elsewhere [24], [39] was developed. The model consisted of lateral skin incisions (2.5 cm in length and 1.25 cm apart) created on the dorsal surface of mice, penetrating the skin, dermis, and underlying adipose tissue. The overlying skin was undermined, and a 0.13-mm-thick silicone sheet was inserted to separate the skin from the underlying tissue bed. The skin was then reapproximated with 6-0 nylon sutures.

### Corneal micropocket assay

The mouse corneal micropocket assay and quantification of neovascularization were performed as described elsewhere [40], using male BALB/c mice (6–10 weeks old). For local delivery, shRNA plasmids (total 10 µg/10 µl per eye) were diluted in phosphate-buffered saline (PBS) and delivered subconjunctivally. The subconjunctival injections were given after hydron pellet implantation.

### Statistical analysis

Data are expressed as mean±SD or mean±SEM. Statistical significance was tested with an unpaired two-tailed Student's *t*-test or analysis of variance (ANOVA). The differences were considered to be significant if *P*<0.05.
